# Comparison between posterior lumbar interbody fusion and posterolateral fusion with transpedicular screw fixation for isthmic spondylolithesis: a meta-analysis

**DOI:** 10.1007/s00402-013-1868-5

**Published:** 2013-10-18

**Authors:** Yong-Ping Ye, Hao Xu, Dan Chen

**Affiliations:** 1 Department of Orthopedics, Fuzhou General Hospital of Nanjing Command, PLA, No. 156 North Xi er huan Road, Fuzhou, 350025 Fujian China; 2 Military Command of Fujian Province, Fuzhou, China

**Keywords:** Posterior lumbar interbody fusion, PLIF, Posterolateral fusion, PLF, Spondylolisthesis, Pain, Fusion rate, Infection rate, Isthmic

## Abstract

**Introduction:**

Primary aim of this study was to compare long-term pain relief and quality of life in adults with isthmic spondylolisthesis (IS) who were treated with posterior lumbar interbody fusion (PLIF) and posterolateral fusion (PLF). Secondary aim was to compare the fusion and infection rates of PLIF- or PLF-treated groups.

**Materials and methods:**

We searched four databases and the cited reference lists of the included studies. Inclusion criteria were pain assessment with visual analog scale (VAS), and clinical studies that compared long-term pain relief of PLF and PLIF-treated adults with IS. Exclusion criteria were use of only one treatment and non-English language.

**Results:**

Three of five included studies used VAS to assess the decline in low back pain, radicular pain, or leg pains in PLF- or PLIF-treated patients during the follow-up periods (0.5–6 years). Long-term pain relief significantly improved in both treatment groups. Pooled differences in mean improvement of Oswestry disability index after the operation revealed no significant difference in pain relief between the PLF and PLIF groups (*P* = 0.856). The five studies together indicated that fusion rate was significantly greater in the PLIF group than that in the PLF group.

**Conclusions:**

The majority of PLIF- and PLF-treated adults with low-grade IS experienced long-term pain relief to a similar extent in most studies. PLIF treatment provided significantly better fusion rates than PLF treatment. This meta-analysis indicates that the use of separate, well-defined scales for pain relief and functional outcomes are needed in studies of PLF or PLIF-treated patients.

## Introduction

Lower back pain of ≥3-month duration afflicts approximately 29 % of adults in the USA [1]. Spondylolisthesis, which can cause low back pain, is the forward or anterior displacement of one vertebra in relation to the adjacent lower vertebra. Types of spondylolisthesis include congenital, isthmic, degenerative, traumatic, pathologic, and postoperative [2]. Although spondylolisthesis can be asymptomatic [3, 4], patients with isthmic spondylolisthesis typically present with low back pain, neurologic symptoms, and/or radicular symptoms. They predominantly affect the L3–S1 region of the vertebrae. Isthmic spondylolisthesis (IS) commonly affects the lumbosacral junction (L5–S1). The incidence of IS is higher in males and ranges from 6 to 8.2 % [4].

Treatment of isthmic spondylolisthesis depends on the severity of symptoms. Patients with physical complaints and mild spondylolisthesis initially are treated with non-surgical modalities including prescriptions for non-steroidal anti-inflammatory drugs (NSAIDs), physical therapy, and modification of their activities that induce pain and rest for 1–2 weeks [3]. These non-surgical treatments combined with anti-lordotic bracing can provide sufficient benefit to more than 75 % of adults with grade I–II spondylolisthesis [3].

After adults fail to respond to 3–6 months of non-surgical treatment, surgical intervention is considered for relief of continual disruptive back pain or radicular pain, loss of nerve function, as well as for symptomatic grade III or greater slip, progressive deformity, and development of cauda equine [3]. The health-related quality of life (QOL) of adults with high-grade spondylolisthesis significantly improves after surgical intervention [5]. Workers have significantly better outcomes than non-working adults in multi-variate analysis [6]. Surgical interventions include decompression, posterior and posterolateral lumbar arthrodesis, and circumferential fusion. Since decompression alone is associated with accelerated disc degeneration and higher rate of slip progression, it is performed mainly in older patients with radicular symptoms [3]. Posterior and posterolateral lumbar arthrodesis now includes fusion of bilateral transverse processes. The reported fusion rate of modern bilateral posterolateral fusion (PLF) is 81–100 % and clinical success rate is 60–98 % [7, 8], regardless of the use of transpedicular fixation [9]. Circumferential fusion theoretically can release the compression on the disc space, increase fusion rate by adding an end plate, and improve correction of the deformity. The three strategies for circumferential fusion include anterior lumbar interbody fusion (ALIF), posterior lumbar interbody fusion (PLIF), and transforaminal lumbar interbody fusion (TLIF). The fusion rate can vary significantly from 74 to 98 % in low-grade adult with IS [8], and short- and long-term follow-up showed significantly improved clinical outcomes in patients who received PLIF (vs. PLF) [10]. However, PLIF requires a longer operating time and can be associated with greater blood loss, more tissue trauma from extensive tissue dissection, more tissue scarring, and risk of misplaced pedicle screws that induce neurological complications [7, 11, 12]. Thus, it is important to elucidate whether the additional risk during the longer PLIF operation provides an improved outcome.

Major outcome criteria for surgical treatment of spondylolisthesis from the patients’ perspective would include pain relief and QOL. In addition, the fusion rate and the incidence of infection with the two procedures may influence which procedure is recommended by the surgeon and staff. Most studies compare small groups and may not have sufficient biostatistical power to detect differences. Thus, the primary objective of this meta-analysis was to determine whether PLF treatments or PLIF treatments were significantly better in improving long-term pain relief and QOL in patients with isthmic spondylolisthesis. The secondary aim included comparisons of the infection rate and the efficiency of spinal fusion of PLIF and PLF.

## Methods

### Literature search

The Medline, Embase, Current Contents, and Cochrane databases were searched using combinations of the key search terms: “posterior lumbar interbody fusion AND posterolateral fusion”; clinical trial; “comparative study and isthmic spondylolisthesis” from inception to August 31, 2012. The grey databases [annual meeting in American College of Orthopedics surgeon, spine surgery, and also neurological surgery (neurosurgery) Journal Club databases] were searched. The reference lists of the included studies and previous systematic reviews were searched for additional relevant articles.

### Included studies

This meta-analysis included randomized controlled trials (RCT), non-RCT, and cohort studies that compared a group of patients with isthmic spondylolisthesis treated with PLF to a group treated with PLIF. The studies also needed to report the pain score before the surgery and at two or more points post-surgery. Fusion rate and infection were also compiled. Exclusion criteria included non-English language, no pain score, and articles reporting results of one technique but not the other.

### Data extraction

Two reviewers independently screened the titles and abstracts of the retrieved articles for adherence to criteria. The potentially relevant full length articles were screened further for the inclusion criteria, and the data were extracted using predetermined forms. Pain score measurements used the visual analog scale (VAS) and functional assessment used the Oswestry disability index (ODI) score. Any disagreements were resolved by a third reviewer.

### Data analysis

Means with standard deviations were calculated for pain score measurement (VAS) and functional measurement (ODI), and were compared between patients with PLF and PLIF. A *χ* ^2^-based test of homogeneity was performed and the inconsistency index (*I* ^2^) statistic was determined. If *I* ^2^ was >50 or >75 %, the trials were considered to be heterogeneous or highly heterogeneous, respectively. If *I* ^2^ was <25 %, the studies were considered to be homogeneous. If the *I* ^2^ statistic (>50 %) indicated heterogeneity existed between studies, a random effects model was calculated. Otherwise, fixed effects models were calculated. Pooled summary statistics of the difference in the mean for the individual studies are shown. Pooled differences in means were calculated and a two-sided *P* value < 0.05 was considered to indicate statistical significance. All analyses were performed using Comprehensive Meta-Analysis statistical software, version 2.0 (Biostat, Englewood, NJ, USA).

## Results

**Fig. 1:**
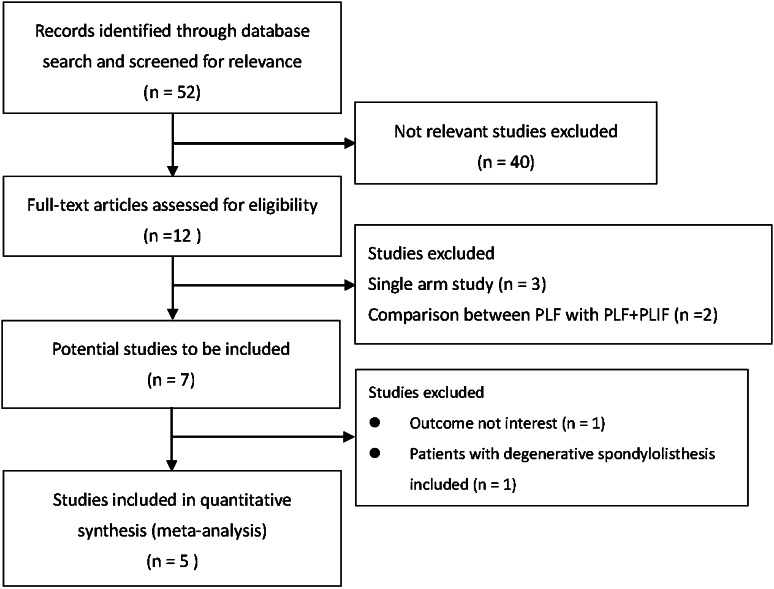
Flow diagram of study selection

Fifty-two articles on PLF and PLIF were retrieved from the databases, and five studies met the initial criteria (Fig. 1). The characteristics of the five published studies [6, 11–13, 15] are summarized in Table 1. The five studies included a total of 389 participants (range 44–163 participants) with 188 adults treated with PLF and 201 adults treated with PLIF. Their demographics showed no significant differences between the two treatment groups in their respective studies (Table 1). Follow-up period ranged from 0.5 years [12] to 1.5 years [13] to 2 years [6, 11] to 5 years [11] to 6 year [13].

**Table 1:** Summary of characteristics of studies included in meta-analysis

First author	Study type	Grades included	Levels	Number of cases^b^	Age^b^	Sex (male, %)^b^	Fusion rate (%)^b^	Pain score measurement (VAS)^b^	Functional measurement (improvement of ODI)^b^	Infection (%)^b^
Farrokhi MR [12]	RCT	N/A	L5–S1	40 vs. 40	49.7 vs. 50.4	25 vs. 23	66.7 vs. 89.1	1 ± 0.98 vs. 1.2 ± 1.58 (radicular pain)	25.34 ± 9.36 vs. 17.00 ± 12.98	2.1 vs. 2.5
Musluman AM [13]	RCT	1-2	L3–S1	25 vs. 25	47.3 vs. 50.6	36 vs. 32	80 vs. 96	1.2 ± 1.04 vs. 1.2 ± 0.76 (leg pain) 2.32 ± 0.9 vs. 1.12 ± 0.66 (back pain)	29.20 ± 6.42 **→** 18.20 ± 3.65 vs. 30.20 ± 5.70 **→** 13.60 ± 1.95	8 vs. 4
Ekman P [6]	NRCT	1–3	L3–L5	77 vs. 86	39 vs. 40	49 vs. 34	N/A	37 vs. 35^a^	N/A	0 vs. 3.5
Dehoux E [15]	NRCT	1–3	N/A	25 vs. 27	42.4 vs. 39.5	56 vs. 51.9	68 vs. 93	N/A	N/A	N/A
Madan S [11]	Retrospective	1–2	L4–S1	21 vs. 23	42.2 vs. 41.2	61.9 vs. 60.9	90.5 vs. 100	N/A	N/A	0 vs. 4.3

*RCT* randomized controlled trial, *NRCT* non-randomized controlled trial, *N/A* not available

^a^Pain index is the mean of two VAS scores for “pain right now” and “worst pain last week”

^b^PLF vs. PLIF

The VAS score for each study measured pain of different variables: VAS recorded the radicular pain in Farrokhi et al. [12], whereas leg pain and back pain were measured in Musluman et al. [13]. The pain index in Ekman et al. [6] was the mean of two VAS scores for “pain right now” and “worst pain last week”, which is different from the standard 10-point VAS. The functional improvement in patients who received PLF vs. PLIF differed significantly in only two of the four studies (Table 1). Greater functional improvement was observed in the PLF-treated group than that in the PLIF-treated group in the study reported by Farrokhi et al. [12]. The leg pain was significantly reduced to a similar extent by both PLF and PLIF procedures in the Musluman study [13]. Whereas the VAS score for the back pain also was significantly reduced by both PLF and PLIF [13], patients treated with PLIF had significantly less low back pain at 1.5–6 years post operation than those treated with PLF at this medical center [13]. The study by Elkman et al. [6] reported that pain was significantly reduced to a similar extent for patients treated with PLF and PLIF after 2 years.

A total of 2–4 studies were included in the meta-analysis for pain (*n* = 2), fusion rate (*n* = 4), and infection rate (*n* = 4) of the five studies that met the inclusion criteria [6, 11–13, 15]. Since Ekman et al. [6] utilized a different pain index and the standard deviation of the improvement of ODI was not available for the two treatment groups, it was not included in the meta-analysis of pain. Both studies included in the meta-analysis on pain [12, 13] reported that the ODI significantly improved in both treatment groups as compared to the pre-operation ODI.

### Disability index

**Fig. 2:**
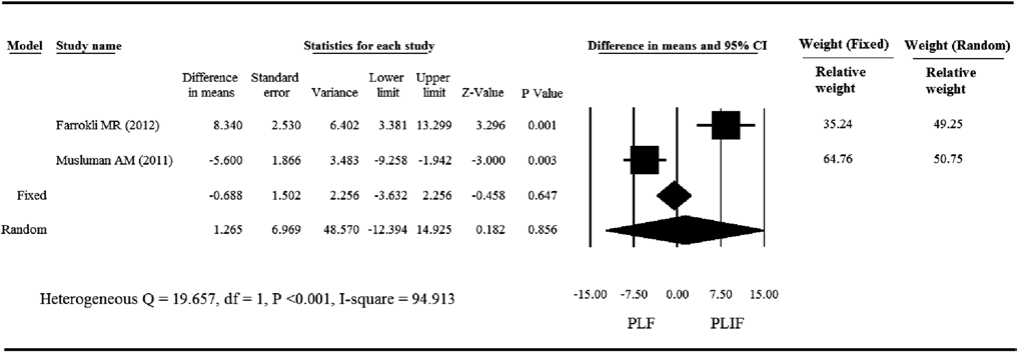
*Forest plot* for improvement of ODI for the studies by Farrokhi et al. [12] and Musluman et al. [13]

The improvement of ODI after the operation was highly heterogeneous between the two treatment groups of these 2 studies (*Q* = 19.675, *I* ^2^ = 94.913 %, *P* < 0.001) (Fig. 2); therefore, a random effects model of analysis was used. Pooled differences in mean improvement of ODI after the operation revealed no significant difference in functional activity between the PLF and PLIF groups (*P* = 0.856). The pooled mean differences in improvement of ODI ranged from −1.265 to 6.969, with the pooled mean differences being 1.265.

### Fusion rate

**Fig. 3:**
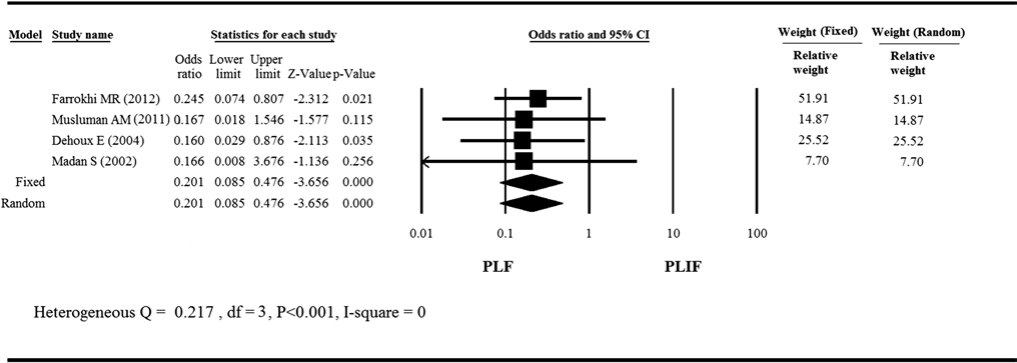
*Forest plot* for fusion rate of four studies by Farrokhi et al. [12], Musluman et al. [13], DeHoux et al. [15], and Madan and Boeree [11]

A meta-analysis of four studies [11–13, 15] compared the fusion rate between the PLF group and the PLIF group (Fig. 3). The fixed effect model was considered as *I* ^2^ = 0 % and the results showed PLF group had lower fusion rate than PLIF group (*P* < 0.001).

### Infection rate

**Fig. 4:**
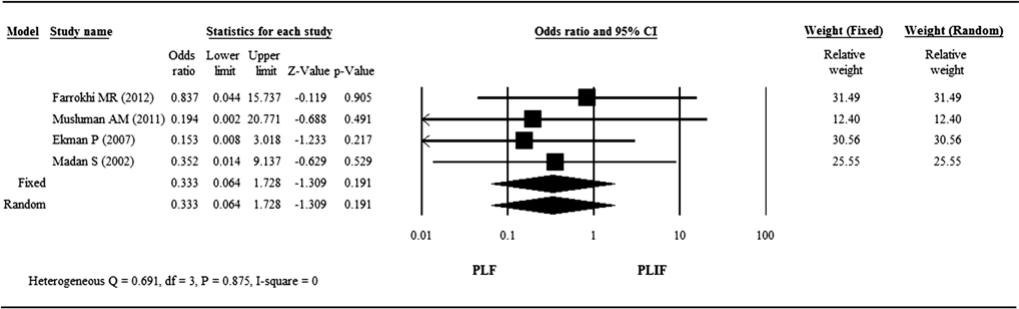
*Forest plot* for infection rate of four studies by Farrokhi et al. [12], Musluman et al. [13], Ekman et al. [6], and Madan and Boeree [11]

A meta-analysis of four studies [6, 11–13] compared the infection rate between the PLF group and the PLIF group (Fig. 4). The fixed effect model was considered as *I* ^2^ = 0 % and the results showed the infection rate after operation was modestly lower in the PLF group in three of the four studies [6, 11, 12], but was not significantly different than the infection rate in the PLIF groups (*P* = 0.191).

Taken together, the five studies indicated that both the PLF and the PLIF techniques provided similar levels of pain relief and functional activity in patients with IS after the long-term follow-up. The rate of infection was modestly less in PLF in three of four studies, but it did not reached significance. The fusion rate of PLIF was significantly greater than that of PLF.

## Discussion

Pain relief and QOL are major outcome criteria for surgical treatment of spondylolisthesis from the patients’ perspective. VAS was used to assess the decline in low back pain, radicular pain, or leg pain in PLF- or PLIF-treated patients during the follow-up periods which ranged from 0.5 to 6 years [6, 12, 13]. Long-term pain relief significantly improved in both the PLF and the PLIF treatment groups in the three studies [6, 12, 13]. While a major aim of this meta-analysis was to determine which treatment group—PLF or PLIF—provided the best long-term pain relief for the treatment of spondylolisthesis, the three studies together indicated that long-term pain relief appeared comparable between the two techniques, in agreement with a recent meta-analysis on the use of PLIF and PLF for the treatment of degenerative lumbar disease [16]. However, it is feasible that one site may be more experienced at one type of circumferential fusion than another type and thus, a specific site may observe better pain relief with PLF [12] than pain relief with PLIF or vice versa [13].

Six additional clinical studies [11, 14, 15, 17–19] and two reviews [7, 8] discussed pain relief by PLF and PLIF surgery for spondylolisthesis, but they did not meet the criteria for inclusion in this meta-analysis on pain from isthmic spondylolisthesis. The pain drawing [11] was used to compare the pain relief from grade 1 or 2 spondylolisthesis by PLF or PLIF, but the groups’ data were not provided. Neurogenic claudication and radicular leg pain were resolved at 100 and 88.5 %, respectively, with both treatments with a minimum follow-up of 2.1 years. Functional improvement assessed by a combination of Oswestry criteria and Deyo’s core questions indicated that a significantly higher percentage of PLIF-treated adults (30 %) had the same or worse functional outcome from pre-operation levels than PLF-treated adults (19 %) [11]. These results agree with the significantly greater improvement in ODI in PLF-treated adults described by Farrokhi et al. [12]. Although PLIF treatment significantly reduces listhesis more than PLF treatment does [11], this greater reduction of listhesis does not correlate with greater pain reduction [11]. Interestingly, 70 % of both PLF- and PLIF-treated adults with spondylolisthesis showed minimal or no disability at long-term follow-up according to the Oswestry criteria [18]. Both treatment groups experienced similar economic and functional improvements according to the Economic and Functional Prolo Scale [18]. However, in a subgroup analysis of the most improved adults with spondylolisthesis, PLIF treatment provided significantly greater outcomes than PLF treatment [18].

The Beaujon scale measures both pain and functional abilities such as walking and QOL [15]: both PLF- and PLIF-treated adults with low-grade slippage obtained similar improvements in pain relief, in agreement with Cheng et al. [14] and Ekman et al. [6]. In contrast, PLIF treatment of adults with grade 2 or 3 slippage had significantly higher pain relief (83 %) than PLF (49 %) [15]; Musluman et al. [13] had reported significantly greater pain relief in PLIF-treated adults with grade 1–2 spondylolisthesis. Similar benefits from PLIF and PLF treatments of adults with spondylolisthesis were obtained at most time points and minimal clinically important differences in the Roland Morris Disability Questionnaire (RMDQ) [17]; but significantly more PLIF-treated adults (96 %) reported less long-term pain on the Low Back Outcome Score than PLF-treated adults with spondylolisthesis (50 %) [17].

The systematic review by Jacobs et al. [7] indicates that PLF, PLIF, and ALIF treatments of adults with low-grade spondylolisthesis provided similar levels of functional outcomes, in agreement with Cheng et al. [14] for degenerative spondylolisthesis, Ekman et al. [6] for IS, Zhou et al. [16] for degenerative lumbar disease, and this meta-analysis for IS. However, Jacobs et al. [7] report similar rates of fusion for PLIF and PLF. The second systematic review [8] suggests that PLF and PLIF achieve similar fusion rates and a successful clinical outcome for most cases of adult low-grade spondylolisthesis. In contrast, both meta-analysis of degenerative lumbar diseases by Zhou et al. [16] and this meta-analysis indicate that the fusion rate was significantly higher in the PLIF-treated group than that in the PLF-treated group.

Limitations of this meta-analysis included the heterogeneous surveys for measuring outcomes and pain relief, the wide variation in follow-up times (0.5 to >6 years), scant reporting of pain as a distinct variable rather than as a part of the clinical outcomes, and the retrospective nature of the case series.

In conclusion, the majority of PLIF- and PLF-treated adults with low-grade isthmic spondylolisthesis experienced long-term relief from low back pain, radicular pain, and leg pain to a similar extent in most studies. The data are consistent with the possibility that PLIF treatment may provide better pain relief and outcomes for adults with higher grade isthmic spondylolisthesis, but further studies are warranted. However, the fusion rate of PLIF-treated group was significantly greater than that of the PLF group. This meta-analysis also indicates that the use of separate and well-defined scales for pain relief and functional outcomes are needed in studies of PLF- or PLIF-treated patients.
